# ZBP1 and TAK1: Master Regulators of NLRP3 Inflammasome/Pyroptosis, Apoptosis, and Necroptosis (PAN-optosis)

**DOI:** 10.3389/fcimb.2019.00406

**Published:** 2019-11-26

**Authors:** R. K. Subbarao Malireddi, Sannula Kesavardhana, Thirumala-Devi Kanneganti

**Affiliations:** Department of Immunology, St. Jude Children's Research Hospital, Memphis, TN, United States

**Keywords:** caspase-1, gasdermin D, MLKL, inflammasome, infection, innate immunity, inflammation, TLR priming

## Abstract

Cell death is central to development, organismal homeostasis, and immune responses. The cell death field has experienced tremendous progress by delineating the molecular programs specific to each of the apoptotic and inflammatory cell death pathways. Moreover, the discovery of the inflammasomes and pyroptosis and necroptosis pathway regulators have provided the genetic basis for the programmed inflammatory cell death pathways. Earlier research highlighted the unique regulation of each of these pathways, but emerging studies discovered co-regulation and crosstalk between these seemingly different cell death complexes. The progress in this area has led to an idea that master regulators play central roles in orchestrating multiple cell death pathways. Here, we provide a brief review of the master regulators, the innate immune sensor ZBP1 and the essential cell survival kinase TAK1, that play vital roles in the regulation of RIPK1/RIPK3–FADD–caspase-8 cell death complex assembly and its versatility in executing Pyroptosis, Apoptosis, and Necroptosis, which we dubbed here as PAN-optosis. Furthermore, we discuss the implications and therapeutic potential of targeting these master regulators in health and disease.

**One Sentence Summary:**

ZBP1 and TAK1 regulate PAN-optosis.

## Introduction

Cell death is fundamentally important for organismal development, homeostasis, and host response to infection. Several cell death pathways have been identified with specific genetically encoded requirements; these have collectively been named programmed cell death pathways (Bergsbaken et al., 2009; Lamkanfi and Dixit, 2010; Pasparakis and Vandenabeele, 2015; Man and Kanneganti, 2016). Apoptosis is the most well-studied form of cell death and was originally considered immunologically silent with major roles in the homeostatic development of multicellular organisms (Kerr et al., 1972; Green et al., 2009). Unlike apoptosis, pyroptosis and necroptosis are considered immunologically active due to their association with membrane lysis and extracellular release of damage-associated molecular patterns (DAMPs), resulting in amplification of inflammation. Receptor-interacting serine/threonine-protein kinase 1 (RIPK1) and RIPK3 play key roles in phosphorylation and activation of the protein mixed lineage kinase domain-like pseudokinase (MLKL) that forms pores in the membrane to execute necroptosis (Feng et al., 2007; Degterev et al., 2008; Cho et al., 2009; He et al., 2009; Zhang et al., 2009; Sun et al., 2012; Zhao et al., 2012; Green, 2019). It is now established that pyroptosis is executed by gasdermin D (GSDMD)-mediated membrane lysis as a result of cleavage by inflammatory caspases to release the functionally active N-terminal fragment (Kayagaki et al., 2015; Shi et al., 2015; Man and Kanneganti, 2016; Van Opdenbosch and Lamkanfi, 2019). The last decade has provided a wealth of information about the molecular details of the inflammasome and inflammatory caspase activation processes that promote pyroptosis and the release of the inflammatory cytokines interleukin (IL)-1 and IL-18; these findings are discussed in detail elsewhere (Kayagaki et al., 2011, 2013; Hagar et al., 2013; Man and Kanneganti, 2016; Van Opdenbosch and Lamkanfi, 2019). As our understanding of these cell death pathways increases, it is becoming clear that in addition to the unique regulation of each of these pathways, there is also significant co-regulation and crosstalk. In this review, we will discuss this co-regulation and the master regulators that play central roles in orchestrating these cell death pathways.

## The Manifestation of PAN-optosis (Pyroptosis, Apoptosis, and Necroptosis)

**Figure 1:**
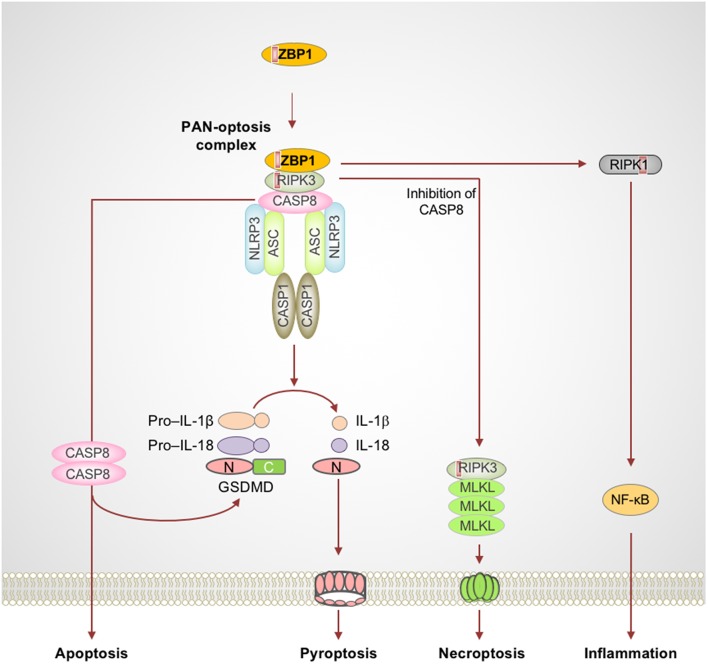
Activation of ZBP1 triggers assembly of signaling complexes to engage PAN-optosis. Z-DNA binding protein 1 (ZBP1) is an innate immune receptor that senses nucleic acids and activates PAN-optosis and inflammation. ZBP1 activation leads to its interaction with receptor-interacting serine/threonine-protein kinase 3 (RIPK3) and recruitment of caspase-8 (CASP8) to form cell death signaling scaffolds. This ZBP1-RIPK3-CASP8 complex engages nucleotide-binding oligomerization domain-like receptor family pyrin domain-containing 3 (NLRP3) inflammasome-dependent pyroptosis, CASP8-mediated apoptosis, and RIPK3-mixed lineage kinase domain-like pseudokinase (MLKL)–driven necroptosis. ZBP1 also induces RIPK1-driven NF-κB activation and inflammation in response to influenza infection. Red boxes within proteins represent the RIP homotypic interaction motif (RHIM) domain. ASC, apoptosis-associated speck-like protein containing a caspase recruitment domain; C, C-terminus; CASP1, caspase-1; GSDMD, gasdermin D; N, N-terminus.

**Figure 2:**
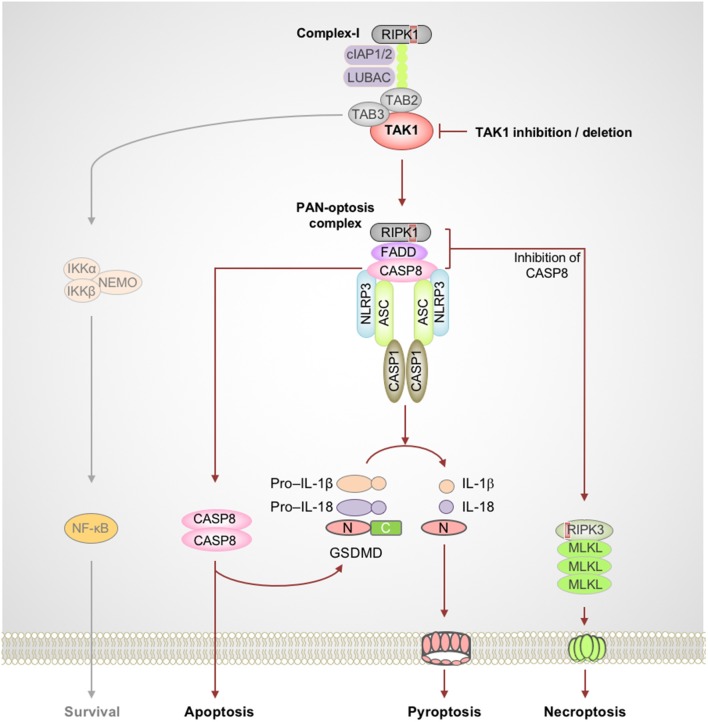
TAK1 acts as a master switch for PAN-optosis quiescence. Genetic deletion or microbial or pharmacological inactivation of transforming growth factor beta-activated kinase 1 (TAK1) function triggers receptor-interacting serine/threonine-protein kinase 1 (RIPK1)-dependent assembly of PAN-optotic cell death complexes. RIPK1 in association with FS7-associated cell surface antigen (Fas)-associated death domain (FADD) and caspase-8 (CASP8) triggers nucleotide-binding oligomerization domain-like receptor family pyrin domain-containing 3 (NLRP3) inflammasome-dependent and CASP8-mediated cleavage of gasdermin D (GSDMD) and the execution of pyroptosis. This complex also engages CASP8-mediated apoptosis, and inhibition of CASP8 activity promotes RIPK3-mixed lineage kinase domain-like pseudokinase (MLKL)–dependent necroptosis. Red boxes within proteins represent the RIP homotypic interaction motif (RHIM) domain. ASC, apoptosis-associated speck-like protein containing a caspase recruitment domain; C, C-terminus; CASP1, caspase-1; cIAP, cellular inhibitor of apoptosis protein; LUBAC, linear ubiquitin chain assembly complex; N, N-terminus; NEMO, NF-κB essential modulator; TAB, TAK1 binding protein.

Cell death plays key roles in infection and immunity, as programmed cell death is often part of the host anti-microbial strategy (Man and Kanneganti, 2016; Jorgensen et al., 2017; Van Opdenbosch and Lamkanfi, 2019). The innate immune system recognizes microbial infections through its pattern recognition receptors (PRRs), which can induce a robust pro-inflammatory immune response and cell death (Lamkanfi and Dixit, 2010; Jorgensen et al., 2017). However, membrane-bound PRRs, such as Toll-like receptors (TLRs) and cytokine receptors such as tumor necrosis factor (TNF) receptor 1 (TNFR1), often show a bias toward induction of the inflammatory immune response through the NF-κB and MAPK pathways, despite having all the principal components required for the induction of cell death (Kawai and Akira, 2007; Li et al., 2010; Thapa et al., 2011; Kaiser et al., 2013; Peltzer et al., 2016; Ting and Bertrand, 2016). As a consequence, several microbes have evolved strategies to target these inflammatory mechanisms. In response, hosts have evolved sophisticated feedback mechanisms which are wired to sense the perturbations in these key nodes of their inflammatory signaling pathways (e.g., the NF-κB and MAPK pathways) and initiate the assembly of multifaceted cell death complexes to drive PAN-optosis to promote inflammation, immune responses, and protection against infection (Mukherjee et al., 2006; Paquette et al., 2012; Philip et al., 2014; Weng et al., 2014; Orning et al., 2018; Sarhan et al., 2018). Pathogen- or pharmacologically mediated obstruction of survival signaling acts as a key danger signal to trigger the assembly of PAN-optotic cell death complexes (Figures 1, 2). Alternatively, monogenic master sensors and regulators have evolved to integrate the complex signals coming from multiple ligands and stimuli associated with live microbial infections. These molecules act as critical sensors of specific microbial infections and as central hubs to trigger both multifaceted cell death in the form of PAN-optosis and inflammatory immune responses. Here we focus on two such key master regulators, Z-DNA binding protein 1 (ZBP1, also called DNA-dependent activator of IFN regulatory factor, DAI, and DLM1) and transforming growth factor beta-activated kinase 1 (TAK1, also called mitogen-activated protein kinase kinase kinase 7, MAP3K7).

## ZBP1 Acts as a Master Switch for PAN-optosis

Influenza A virus (IAV) is a single-stranded RNA virus which infects millions worldwide and poses a major threat to public health. Early reports provided the first evidence of a crucial role for nucleotide-binding oligomerization domain (NOD)-like receptor (NLR) family pyrin domain-containing 3 (NLRP3) in sensing IAV infection and dsRNAs to assemble the inflammasome complex via the adapter protein apoptosis-associated speck-like protein containing a caspase recruitment domain (ASC) and drive caspase-1–mediated inflammation (Kanneganti et al., 2006a,b). Furthermore, IAV-induced activation of the NLRP3 inflammasome controls viral spread and protects against lung damage, promoting host-protective immune responses (Allen et al., 2009; Thomas et al., 2009). While IAV RNA recognition is known to induce inflammation and cell death, the identity of innate sensors that mediate programmed inflammatory cell death and inflammation were not known for many years. ZBP1 was recently identified as an innate sensor of IAV infection (Kuriakose et al., 2016; Thapa et al., 2016; Kesavardhana et al., 2017). Upon sensing IAV infection, ZBP1 activation leads to NLRP3 inflammasome activation and PAN-optosis (Kuriakose et al., 2016; Kesavardhana et al., 2017). ZBP1 also critically regulates the IAV-induced pro-inflammatory cytokine response (Kuriakose et al., 2016; Kesavardhana et al., 2017).

ZBP1 is one of the RIP homotypic interaction motif (RHIM)-containing proteins and is known to activate necroptosis via RIPK3, another RHIM-containing protein (Rebsamen et al., 2009; Upton et al., 2012; Kuriakose and Kanneganti, 2018). ZBP1 is unique among cell death proteins since it contains a RHIM domain to mediate cell death and also a Zα domain which binds Z-nucleic acids (Rebsamen et al., 2009; Kuriakose and Kanneganti, 2018). Thus, ZBP1 not only engages cell death signaling but is also capable of sensing aberrant nucleic acids and instructing the assembly of cell death signaling scaffolds. Recent studies suggest that ZBP1 directly senses IAV infection and facilitates RIPK3 and caspase-8 activation (Kuriakose et al., 2016; Kesavardhana et al., 2017). ZBP1, in association with RIPK3 and caspase-8, promotes assembly of the signaling complexes that mediate PAN-optosis (Kuriakose et al., 2016; Kesavardhana et al., 2017; Kuriakose and Kanneganti, 2018) (Figure 1). Deletion of ZBP1 completely abolishes IAV-induced NLRP3 inflammasome activation and the release of leaderless cytokines. ZBP1-mediated PAN-optosis after IAV infection is dependent on the RIPK3–caspase-8 complex but independent of RIPK1. However, ZBP1 is essential for the RIPK1-dependent pro-inflammatory cytokine response during IAV infection, although ZBP1 and RIPK1 do not directly interact in endogenous conditions. In addition, ZBP1 mediates necroptosis and inflammation when the RIPK1 RHIM domain is mutated and causes perinatal lethality in *Ripk1*^RHIM/RHIM^ mice (Lin et al., 2016; Newton et al., 2016). ZBP1 also promotes the activation of RIPK3 in the absence of RIPK1-RHIM function. These findings establish that, in physiological settings, RIPK1-RHIM function counteracts ZBP1-mediated cell death and inflammation for organismal homeostasis. Based on these findings, it is possible that a putative signaling complex might control RIPK1-mediated inhibition of ZBP1 function, independent of the canonical ripoptosome complex which contains RIPK1 as a core scaffolding protein.

ZBP1 activation requires upstream activation of other nucleic acid sensors. Recent studies suggest that recognition of IAV RNA by the RIG-I receptor initiates type I IFN/IFNAR signaling to license ZBP1 upregulation and activation (Kesavardhana et al., 2017). These studies found that ZBP1 senses the viral ribonucleoprotein complexes (vRNPs) of IAV that are generated during replication to activate cell death pathways. How ZBP1 is activated in the absence of RIPK1-RHIM function and what the endogenous nucleic acid ligands that activate ZBP1 are still remain as unexplored questions in this field.

## TAK1 Acts as a Master Regulator for PAN-optosis Quiescence

The TNF family of cytokine receptors plays important roles in inflammation and cell death and shapes the nature of host innate and adaptive immune responses. Often, the default outcome of triggering these cell surface receptors is cell survival and cytokine production. In immune cells, the TNF-TNFR1 complex proximal to the membrane enables the formation of the RIPK1-containing complex-I which promotes prosurvival and inflammatory responses (Peltzer et al., 2016; Ting and Bertrand, 2016). The inactivation of essential complex-I components such as TAK1 results in the assembly of a cytosolic ripoptosome-like cell death complex with the potential to drive multifaceted cell death, PAN-optosis. The inhibitory phosphorylation of RIPK1 by TAK1 is critical to restrict its activation and blocks the spontaneous activation of PAN-optosis. The PAN-optotic death complex containing RIPK1, FS7-associated cell surface antigen (Fas)-associated death domain (FADD), and caspase-8 acts as the core and is assembled through their death domain (DD) interactions. This complex promotes FADD–caspase-8–dependent apoptosis through the activation of effector caspase-3 and -7 and promotes necroptosis by RIPK3-mediated phosphorylation of MLKL (Figure 2). Recent studies demonstrated that this ripoptosome-like complex also plays a key role in activation of the NLRP3 inflammasome and pyroptosis when TAK1 is inhibited (Malireddi et al., 2018, in press; Orning et al., 2018; Sarhan et al., 2018).

The essential role of TAK1 in innate immunity against microbes resulted in the evolution of microbial inhibitors that block its function to promote immune evasion (Mukherjee et al., 2006; Paquette et al., 2012; Philip et al., 2014; Weng et al., 2014; Malireddi et al., 2018; Orning et al., 2018; Sarhan et al., 2018). For example, the intracellular bacteria *Yersinia* has evolved to produce the toxin YopJ that inactivates TAK1 (Mukherjee et al., 2006; Paquette et al., 2012). However, feedback mechanisms in the host sense the perturbation of TAK1 signaling as an intracellular pathogenic insult and trigger RIPK1 kinase activity-dependent PAN-optosis (Malireddi et al., 2018). Furthermore, recent studies focused on pharmacological- or pathogen-based inhibition of TAK1 have revealed a direct role for caspase-8, which is activated in the RIPK1–FADD–caspase-8 complex, in driving GSDMD cleavage and pyroptosis, independent of caspase-1 (Orning et al., 2018; Sarhan et al., 2018). Additionally, it was shown that microbial priming bypasses the requirement for RIPK1 kinase activity to drive PAN-optosis when TAK1 is inactivated (Malireddi et al., in press). Together, these findings are particularly important since they demonstrate the versatility of this cell death complex and provide the proof-of-concept for the existence of the PAN-optotic complex. Moreover, mice deficient in components of the PAN-optotic complex were shown to be susceptible to *Yersinia* infection (Philip et al., 2014; Weng et al., 2014), demonstrating the functional importance of this cell death complex.

## Converging Mechanisms of Programmed PAN-optosis

Recent research focused on inflammasomes and inflammatory caspases led to the discovery of extensive crosstalk between the apoptotic and inflammatory cell death pathways. Studies focused on the RHIM domain-containing proteins (RIPK1 and RIPK3) and their crosstalk with FADD–caspase-8–mediated pathways improved our understanding of the apoptotic and necroptotic pathways and their intricate regulatory mechanisms (Holler et al., 2000; He et al., 2009; Zhang et al., 2009; Gunther et al., 2011; Kaiser et al., 2011; Oberst et al., 2011; Peter, 2011; Welz et al., 2011; Wrighton, 2011). In parallel, several studies of innate immune receptors showed unique and overlapping roles in assembling a diverse array of caspase-activating inflammasomes and activating pyroptotic cell death (Kesavardhana and Kanneganti, 2017; Karki and Kanneganti, 2019). Of these, the NLRP3 inflammasome has emerged as an extremely versatile sensor of stress that responds to a wide range of microbial and damage-promoting cytotoxic insults (Kesavardhana and Kanneganti, 2017; Karki and Kanneganti, 2019). Generation and characterization of NLRP3-deficient mice provided the first concrete and genetic evidence for its importance in sensing bacterial and viral components and in the induction of inflammatory caspase-1 activation and maturation of pro–IL-1β and pro–IL-18 (Kanneganti et al., 2006a,b; Mariathasan et al., 2006; Sutterwala et al., 2006). More recently, studies have revealed an unexpected amount of crosstalk between pyroptosis and apoptosis and necroptosis. While early studies indicated that caspase-8 could contribute to inflammatory functions by driving maturation of inflammatory IL-1 cytokines (Maelfait et al., 2008; Bossaller et al., 2012; Gringhuis et al., 2012; Vince et al., 2012; Man et al., 2014), Gurung et al. provided the first definitive evidence for direct crosstalk between the apoptotic and pyroptotic components. This study demonstrated that FADD and caspase-8 are crucial for canonical and non-canonical inflammasome activation and inflammatory cell death (Gurung et al., 2014). Moreover, ASC, an important adapter for inflammasome assembly, was also shown to be recruited to the caspase-8–containing cell death complex (Van Opdenbosch et al., 2017; Lee et al., 2018). More recent studies focused on the innate immune sensor ZBP1 and the essential kinase TAK1 have further strengthened our understanding of the versatility of the RIPK1/RIPK3–FADD–caspase-8 cell death complex and provided a strong foundation for the emerging concept of PAN-optosis (Kuriakose et al., 2016; Malireddi et al., 2018; Orning et al., 2018; Sarhan et al., 2018) (Figures 1, 2). Furthermore, defects in TAK1-associated cell survival signaling molecules also trigger similar multifaceted cell death pathways and promote inflammatory immune responses (Gerlach et al., 2011; Vince et al., 2012; Dondelinger et al., 2013, 2015; Lawlor et al., 2015; Moriwaki et al., 2015; Peltzer et al., 2018; Zhang et al., 2019). This newly proposed concept of PAN-optosis should encourage further future attempts to expand our understanding of the co-regulation of multifaceted cell death complexes during cell death and inflammation and the relevance of this co-regulation to health and disease.

## PAN-optosis in Inflammatory Diseases

The studies focused on caspases, cell death, and inflammation have demonstrated that there is a constant competition between the host and microbes to exploit mechanisms of cell death and inflammation to optimize their own survival, and the outcome is not always beneficial to the host (Lamkanfi and Dixit, 2010; Malireddi and Kanneganti, 2013). Loss- or gain-of-function mutations in the key components of different cell death pathways result in dysregulated immune responses to infection or promote the development of debilitating inflammatory diseases. For example, liberation of constraints on ZBP1 function leads to autoinflammation and perinatal lethality in mice, indicating manifestations of PAN-optosis in pathophysiology and organismal development (Lin et al., 2016; Newton et al., 2016). Consistent with its role in driving robust cell death and inflammation, inhibition of TAK1 can lead to unwanted activation of PAN-optosis, predisposing an individual to the development of inflammatory diseases. In support of this concept, genetic deletion of TAK1 in mice was shown to result in spontaneous cell death and embryonic lethality (Sato et al., 2005; Shim et al., 2005). Moreover, mutations resulting in reduced TAK1 function in humans and mice lead to loss of immune homeostasis and myeloid-proliferation syndromes (Ajibade et al., 2012; Eftychi et al., 2012; Lamothe et al., 2012). Interestingly, an aging-associated reduction of TAK1 expression was shown to cooperate with other genetic risk factors to promote the RIPK1-dependent onset of neuroinflammation and the development of amyotrophic lateral sclerosis (ALS) in humans (Xu et al., 2018). Furthermore, loss of TAK1 in myeloid cells in mice results in hypersusceptibility to LPS shock and enhanced neutrophilic proliferation, supporting a crucial role for TAK1-mediated inhibition of PAN-optosis in preventing inflammation and maintaining immune homeostasis (Ajibade et al., 2012; Eftychi et al., 2012; Malireddi et al., 2018; Sanjo et al., 2019). These studies support that ZBP1 and TAK1 act as master regulators of multiple cell death pathways and are essential to control cellular homeostasis and PAN-optosis and to prevent inflammatory pathophysiology.

Similarly, genetic deficiency of the NF-κB and MAPK components suggests a role for the necrosome components RIPK1 and RIPK3 in promoting NLRP3 inflammasome activation, triggering multiple forms of cell death and the development of inflammatory diseases (Ikeda et al., 2011; Matmati et al., 2011; Tokunaga et al., 2011; Vince et al., 2012; Kumari et al., 2014; Rickard et al., 2014; Vande Walle et al., 2014; Gurung et al., 2015; Xu et al., 2018; Peltzer and Walczak, 2019; Polykratis et al., 2019; Yuan et al., 2019). Similarly, disease in *Pstpip2*^cmo^ mice, which have a missense mutation in the proline-serine-threonine phosphatase-interacting protein 2 (*Pstpip2*) gene that results in debilitating inflammatory arthritis similar to that of patients with chronic multifocal osteomyelitis (cmo), is rescued when caspase-8 is inactivated in combination with either caspase-1 or the NLRP3 inflammasome (Ferguson et al., 2006; Grosse et al., 2006; Lukens et al., 2014; Gurung et al., 2016). These observations indicate that the inflammasome, caspase-1, and caspase-8 have redundant roles and potentially become activated in a multifaceted PAN-optotic complex to promote the inflammatory disease in *Pstpip2*^cmo^ mice. Together, these findings support the convergent evolution and shared mechanism of PAN-optosis with potential implications in the development of inflammatory disease.

## Summary and Future Perspectives

Recent work has resulted in tremendous progress in our understanding of cell death pathways with the discovery of novel regulators and the crosstalk between pyroptosis, apoptosis, and necroptosis. These studies also revealed the existence of master regulators such as ZBP1 and TAK1 that act as key signaling nodes for cell death. Recent progress in this area has also revealed an intimate connection between the RIPK1/RIPK3–FADD–caspase-8 signaling complex and the execution of PAN-optosis. It is clear that the PAN-optotic death complexes activated by the master regulators are multifaceted in nature with the potential to execute diverse forms of caspase-mediated cell death and promote necroptosis when caspases are inhibited. Together, these findings lay the foundation for the concept of master regulators controlling multifaceted cell death, PAN-optosis. Future studies should reveal additional mechanisms of PAN-optosis to better understand its evolutionary relevance and significance in health and disease. Further understanding of the master regulators of PAN-optosis may also allow us to develop superior therapeutic approaches to target cancer, infection, and inflammatory diseases.

## Author Contributions

RM, SK, and T–DK drafted and edited the review.

## Conflict of Interest

The authors declare that the research was conducted in the absence of any commercial or financial relationships that could be construed as a potential conflict of interest.
